# Engineering Robust Ag‐Decorated Polydopamine Nano‐Photothermal Platforms to Combat Bacterial Infection and Prompt Wound Healing

**DOI:** 10.1002/advs.202106015

**Published:** 2022-02-22

**Authors:** Xiaoliang Qi, Yijing Huang, Shengye You, Yajing Xiang, Erya Cai, Ruiting Mao, Wenhao Pan, Xianqin Tong, Wei Dong, Fangfu Ye, Jianliang Shen

**Affiliations:** ^1^ State Key Laboratory of Ophthalmology Optometry and Vision Science School of Ophthalmology and Optometry School of Biomedical Engineering Wenzhou Medical University Wenzhou Zhejiang 325027 China; ^2^ School of Chemical Engineering Nanjing University of Science and Technology Nanjing Jiangsu 210094 China; ^3^ School and Hospital of Stomatology Wenzhou Medical University Wenzhou Zhejiang 325027 China; ^4^ Wenzhou Institute University of Chinese Academy of Sciences Wenzhou Zhejiang 325000 China; ^5^ Oujiang Laboratory (Zhejiang Lab for Regenerative Medicine, Vision and Brain Health) Wenzhou Zhejiang 325001 China

**Keywords:** Ag‐decorated polydopamine nanoparticles, antibacterial therapy, photothermal agent, photothermal conversion efficiency, polysaccharide hydrogel

## Abstract

Polydopamine (PDA) nanoparticles have emerged as an attractive biomimetic photothermal agent in photothermal antibacterial therapy due to their ease of synthesis, good biodegradability, long‐term safety, and excellent photostability. However, the therapeutic effects of PDA nanoparticles are generally limited by the low photothermal conversion efficiency (PCE). Herein, PDA@Ag nanoparticles are synthesized via growing Ag on the surface of PDA nanoparticles and then encapsulated into a cationic guar gum (CG) hydrogel network. The optimized CG/PDA@Ag platform exhibits a high PCE (38.2%), which is more than two times higher than that of pure PDA (16.6%). More importantly, the formulated CG/PDA@Ag hydrogel with many active groups can capture and kill bacteria through effective interactions between hydrogel and bacteria, thereby benefiting the antibacterial effect. As anticipated, the designed CG/PDA@Ag system combined the advantages of PDA@Ag nanoparticles (high PCE) and hydrogel (preventing aggregation of PDA@Ag nanoparticles and possessing inherent antibacterial ability) is demonstrated to have superior antibacterial efficacy both in vitro and in vivo. This study develops a facile approach to boost the PCE of PDA for photothermal antibacterial therapy, providing a significant step forward in advancing the application of PDA nano‐photothermal agents.

## Graphical abstract

This work presents a facile and convenient strategy to boost the photothermal conversion efficiency of polydopamine (PDA) nanomaterials via growing Ag nanoparticles on the surface of PDA and then encapsulated into a cationic guar gum hydrogel network. The resultant system possesses robust photothermal performance that effectively kills bacteria and promotes wound healing.

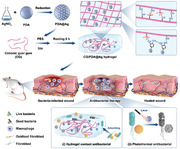

## 1 Introduction

Infectious diseases induced by pathogenic bacteria range from mild cutaneous infections to life‐threatening toxinoses, which have caused substantial economic losses and considerable threats to global health.^[^ ^1^, ^2^ ^]^ Antibiotics have been widely used as the most common treatment for bacterial infections.^[^ ^3^, ^4^ ^]^ However, the improper utilization, especially the overuse of antibiotics, has led to the appearance of many multidrug‐resistant bacteria.^[^ ^5^ ^]^ Therefore, developing robust alternative therapeutics to eradicate bacterial infections without inducing multidrug‐resistance (MDR) has become an emergent goal.^[^ ^6^ ^]^ Recently, effective methods such as chemodynamic therapy (CDT), photodynamic therapy (PDT), sonodynamic therapy (SDT), and photothermal therapy (PTT) have been applied to managing bacterial infection. Among these antimicrobial therapies, PTT that kills bacteria by physical heat has attracted widespread attention due to its fewer side effects, good controllability, minimal invasiveness, high selectivity, and less potential to induce MDR microbes.^[^ ^7^ ^]^ In particular, near‐infrared (NIR) light‐responsive PTT has been widely investigated because of NIR light's excellent tissue penetration depth.^[^ ^8^, ^9^ ^]^ During PTT, the energy of NIR light is converted into heat through photothermal agents (PTAs) after irradiation at target sites, leading to bacteria death by damaging bacterial cell membranes and causing protein denaturation.

Various kinds of inorganic materials (transitional metal dichalcogenide, gold nanoparticles, palladium nanosheets, and carbon nanomaterials) and organic nanoparticles (porphyrins, cyanines, polypyrrole, polyaniline, and polydopamine) are being developed as PTAs for PTT.^[^ ^9^ ^]^ Polydopamine (PDA), a significant naturally occurring melanin analogue, is one of the most promising PTAs due to its ease of fabrication, excellent photostability, favorable biodegradability, and superior biocompatibility.^[^ ^10^, ^11^, ^12^, ^13^ ^]^ PDA nanomaterials with various morphologies (nanotubes, nanospheres, and nanosheets) and different sizes (ranging from 20 to 500 nm) have been designed for PTT in recent years.^[^ ^14^, ^15^, ^16^, ^17^ ^]^ However, there are mainly two defects involved in the currently developed PDA nano‐PTAs. One is that, like many other organic nanomaterials, the photothermal conversion efficiency (PCE) of PDA is relatively low (about 20%) under 808 nm NIR laser irradiation and even lower PCE at longer wavelengths, which is mainly attributed to incomplete nonradiative transition and then induces weak NIR absorbability.^[^ ^18^, ^19^ ^]^ The other is that PDA nanomaterials are easily aggregated because of the existence of a large number of active moieties on the surface of nano‐PDA like catechol, primary amine, and secondary amine groups. The emergence and aggregation of PDA nanomaterials will decrease light absorbance efficiency, eventually resulting in reduced PCE. These two defects severely hinder the practical applications of PDA‐based nano‐PTAs. Therefore, how to increase the light absorption efficiency and reduce the aggregation of nanomaterials simultaneously is one of the main obstacles to engineering high photothermal performance PDA‐based PTAs for treating bacterial infections.

To boost the PCE of PDA nanomaterials, extensive efforts have been made from two aspects involving augmenting light absorption efficiency in the NIR region by reducing nonradiative transition and promoting the dispersion of nanomaterials by introducing an appropriate carrier. For example, Cheng and co‐workers found that the photothermal performance of PDA could be improved by in situ growing metal nanoparticles on the surface of PDA, which was attributed to the accelerated charge transfer efficiency and then induced enhanced nonradiative transition.^[^ ^20^ ^]^ Zeng et al. demonstrated that PDA aggregation decreased when loading PDA in a hydrogel matrix, achieving high PTT efficacy.^[^ ^10^ ^]^ These results remind us that PDA decorated with metal nanoparticles and subsequently encapsulated in a hydrogel scaffold may be conducive to the photothermal performance improvement of PDA.

**Figure 1:**
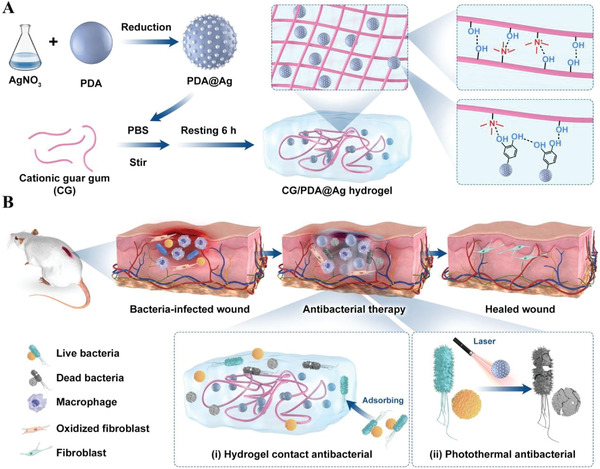
Schematic illustrating the fabrication of CG/PDA@Ag hydrogel and its application as a photothermal antibacterial platform for wound dressing. A) Using a simply one‐pot mixing injection approach, B) we facilely constructed a robust hydrogel dressing that can promote wound healing by synergistic antibacterial therapy like hydrogel contact and photothermal effect.

To validate the above hypothesis, herein, we constructed a biocompatible polysaccharide hydrogel loading with PDA@Ag nanoparticles for application in focal infection treatment. As displayed in **Figure** **1**, Ag nanoparticles growing on the surface of PDA nanoparticles were achieved by the reduction‐deposition approach. The photothermal converting rate was significantly improved through Ag modification from 16.6% (PDA) to 36.1% (PDA@Ag). Subsequently, PDA@Ag nanoparticles were embedded into the polysaccharide (cationic guar gum, CG) network to prepare the CG/PDA@Ag hydrogel by a facile one‐pot blending method. With the help of a hydrogel carrier, on the one hand, PDA@Ag nanoparticles can be uniformly dispersed without agglomeration, and the PCE of the designed CG/PDA@Ag system was further increased from 36.1% to 38.2%. On the other hand, the CG polysaccharide hydrogel with abundant functional hydroxyl and quaternary ammonium groups, which can nonselectively capture and kill some Gram‐positive and Gram‐negative bacteria by interacting with bacteria through electrostatic forces, Van der Waals forces, and hydrophobic interactions, can provide an additional antibacterial effect. Antibacterial tests demonstrated that this composite CG/PDA@Ag hydrogel possessed broad‐spectrum antibacterial properties against *Escherichia coli* (*E. coli*, 99.9%) and *Staphylococcus aureus* (*S. aureus*, 99.8%) in vitro. Further results indicated CG/PDA@Ag hydrogel also displayed outstanding bactericidal activity in a rat wound infection model in vivo. As far as we know, this is the first report on applying CG/PDA@Ag composite hydrogel in the field of antibacterial therapy. Our work provides new ideas for constructing a robust PDA‐based photothermal system for antibacterial therapy and wound healing.

## 2 Results and Discussion

### 2.1 Fabrication and Characterization of PDA@Ag Nanoparticles

PDA@Ag nanoparticles were facilely prepared in this work via a one‐step redox reaction approach, as illustrated in Figure 1. In the present investigation, the deposition of Ag nanoparticles was chosen because Ag nanoparticles possessed good photoelectric performance that may improve the PCE of designed nanoparticles.^[^ ^21^ ^]^ In the fabrication process, the diverse functional groups (such as dihydroxyphenyl groups) on the surface of PDA actively participated in Ag^+^ ions reduction‐deposition, which can reduce the Ag^+^ into Ag^0^, forming coordination linkages with Ag nanoparticles.^[^ ^22^ ^]^

**Figure 2:**
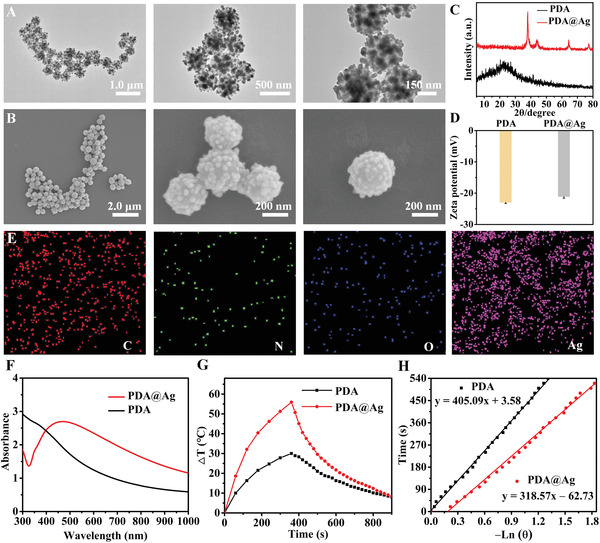
Characterization of PDA@Ag nanoparticles. A) TEM images, B) SEM images, C) XRD patterns, and D) zeta potentials of PDA and PDA@Ag nanoparticles. E) SEM elemental mapping images of C, N, O, and Ag of PDA@Ag nanoparticles. F) UV–vis–NIR absorption spectra, G) photothermal effects, and H) plot fitting of cooling time versus the negative natural logarithm of the driving force temperature during the cooling phase of PDA and PDA@Ag nanoparticles.

The morphology of the designed PDA@Ag nanoparticles was first characterized by transmission electron microscopy (TEM) (**Figure** **2**A) and scanning electron microscopy (SEM) (Figure 2B). The Ag nanoparticles possessing an average diameter of 30 nm were relatively uniformly distributed on the surface of the PDA nanoparticles, and the Ag‐decorated PDA nanoparticles presented a uniform spherical structure with a diameter of 290 nm. Then, the powder X‐ray diffraction (XRD) pattern of PDA@Ag nanoparticles was compared with that of PDA nanoparticles. As shown in Figure 2C, PDA exhibited a broad halo at *2θ* = 24.2°, suggesting its amorphous nature. For the XRD pattern of PDA@Ag, the presence of metallic Ag can be observed by the Ag(111) at 37.9°, Ag(200) at 44.1°, Ag(220) at 64.3°, and Ag(311) at 77.2°.^[^ ^23^ ^]^ Furthermore, zeta potentials of nanoparticles changed from −23 mV (PDA) to −21.1 mV (PDA@Ag) because the reduction of Ag^+^ ions into metallic Ag° consumed a certain amount of negatively charged groups of PDA (Figure 2D). Afterward, SEM‐assisted element mapping images demonstrated the existence of homogeneously distributed C, N, O, and Ag elements, confirming the successful fabrication of PDA@Ag nanoparticles (Figure 2E). Next, we checked the UV–vis–NIR absorption spectra of as‐prepared PDA and PDA@Ag nanoparticles. Silver nanoparticles are widely recognized for having a size‐dependent surface plasmon resonance band at around 440 nm that can be detected using UV–vis–NIR spectroscopy.^[^ ^24^ ^]^ As expected, in contrast to the PDA, the absorption of PDA@Ag at 808 nm was much higher, which may be due to the UV absorption brought by Ag, suggesting that PDA@Ag was more suitable for PTT (Figure 2F). Similar spectral absorption enhancement from deposition of Ag nanoparticles was reported by other researchers.^[^ ^25^ ^]^ Notably, the photothermal conversion efficiency (*η*) of PDA and PDA@Ag at 808 nm was calculated to be 16.6% and 36.1% (Figure 2G,H), according to Equations (1)–(3), revealing that the photothermal performance of the hybrid system was significantly improved after the Ag deposition^[^ ^21^ ^]^

(1) $$\eta =\frac{hA\left(T_{\max,\mathrm{sam}}-T_{\max,\mathrm{water}}\right)}{I\left(1-10^{-A_{808}}\right)}$$

(2) $$t=\frac{\sum_{i}m_{i}C_{P,i}}{hA}\;In\theta$$

(3) $$\theta =\frac{T-T_{\mathrm{sur}}}{T_{\max}-T_{\mathrm{sur}}}$$

where *T*_max,sam_ represents the maximum temperature induced by samples; *T*_max,water_ represents the maximum temperature induced by water; *I* represents the laser power; *A*_808_ represents the absorbance of samples aqueous solution at 808 nm; *m*_i_ represents the weight of water; *C*_p,i_ represents the heat capacity of water; and *θ* represents the system constant.^[^ ^26^ ^]^

### 2.2 Fabrication and Characterization of CG/PDA@Ag Hydrogels

To endow the PDA@Ag nanoparticles with good biocompatibility and improved photothermal performance, three CG/PDA@Ag hydrogels (CPA1, CPA2, and CPA3) were synthesized by an environmentally friendly one‐pot mixing method. Briefly, PDA@Ag nanoparticles were added into the cationic guar gum (CG) precursor. This mixture quickly experienced sol‐gel transition due to the multiple noncovalent interactions between cationic CG networks and anionic PDA@Ag nanoparticles, including hydrogen bonds, electrostatic forces, and *π*–*π* stacking. These recoverable noncovalent bonds can endow the resultant hydrogel with injectability and self‐healing ability, providing more convenience for the practical photothermal application of CG/PDA@Ag hydrogel. In addition, the color of the hydrogel changed from white to black after introducing PDA@Ag nanoparticles into the CG matrix (Figure S1, Supporting Information).

The structure information of CG/PDA@Ag (CPA2) hydrogels was first investigated by SEM. As revealed in Figure S2A,B (Supporting Information), the pure CG hydrogel preserved a porous interconnected structure. Following PDA@Ag loading, the hybrid hydrogel mainly displayed a honeycomb‐like structure, and higher magnification verified that PDA@Ag was indeed uniformly present inside the hydrogel in the form of nanoparticles (Figure S2C,D, Supporting Information). Subsequently, the chemical structure of the resultant hydrogels was identified by Fourier transform infrared (FTIR) (Figure S3, Supporting Information). Both pure CG hydrogel and hybrid CG hydrogel had O—H, C—H, and sugar ring vibrations at about 3300, 2918, and 1648 cm^−1^.^[^ ^27^, ^28^ ^]^ Interestingly, after the incorporation of nanoparticles, a redshift occurred in hybrid CG hydrogel (from 3321 to 3299 cm^−1^) may be due to the established hydrogen bonds of the polysaccharide network being partially destroyed by the newly formed interactions between CG and PDA@Ag like electrostatic forces, indirectly confirming the successful integration of PDA@Ag nanoparticles with CG matrix. Then, the thermal stability of the hydrogel structure was explored (Figure S4, Supporting Information). The remaining weight of CPA2 hydrogel was slightly higher than that of pure CG hydrogel at the terminated temperature of 600 °C, indicating the improved thermal stability of hybrid hydrogel due to the existence of PDA@Ag nanoparticles. Afterward, the dynamic swelling ratio of the hydrogels was studied by immersing the hydrogels in PBS until reaching swelling equilibrium (Figure S5, Supporting Information). Equilibrium swelling ratios of CG and CPA2 were determined to be 20.2 and 17.9, respectively, attributed to increased hydrogel crosslinking density.^[^ ^29^ ^]^ The possible reason was that more noncovalent interactions such as electrostatic interactions appeared in hydrogel after incorporating PDA@Ag nanoparticles into CG matrix, leading to densely hydrogel networks and thereby hindering the diffusion of water into the hydrogel. At the same time, water in CPA2 hydrogel was difficult to escape from the compact crosslinked network compared with that of the pure CG hydrogel, as proved by the water retention test. In other words, compared with pure CG hydrogel without PDA@Ag nanoparticles, the designed CPA2 hydrogel had better water retention capacity that can maintain the moist environment needed for wound repair for a more extended period (Figure S6, Supporting Information). Additionally, after soaking in PBS solution for 6 days, CPA2 hydrogel could be thoroughly degraded (Figures S7 and S8, Supporting Information). This feature ensured that when used as a wound dressing, CPA2 hydrogel had the potential to be totally degraded and therefore did not cause secondary removal damage to the wound.

**Figure 3:**
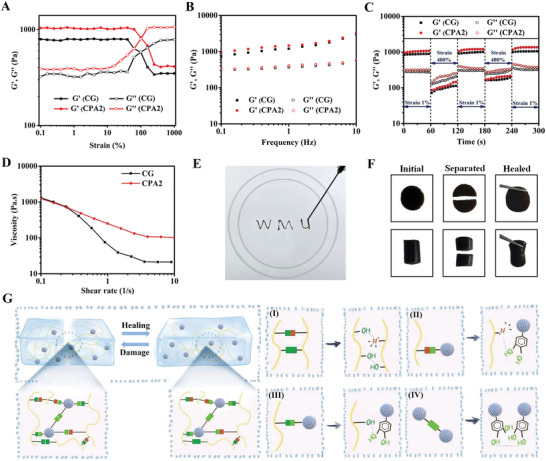
Mechanical properties and self‐healing mechanism of prepared hydrogels. A–D) Rheological performance: strain sweep, frequency sweep, dynamic step–strain, and viscosity measurements of CG and CPA2 hydrogels, respectively. E) The injectable CPA2 hydrogel. F) Photographs of the self‐healing process of CG/PDA@Ag hydrogel. G) Schematic diagram of the possible self‐healing mechanism of CG/PDA@Ag hydrogel.

Generally, hydrogels served as wound dressings need to have specific mechanical properties to meet the needs of different wounds like irregular and deep wounds. For example, hydrogel dressings had the self‐healing ability to cope with the pressure at the wound sites originated from body movement, which will improve their security of use and prolong the service time,^[^ ^27^ ^]^ whereas hydrogel dressings that can be used by injection are more convenient in practice. Here, the mechanical properties of fabricated hydrogels were systematically investigated. First, the strain sweep assay demonstrated that the hydrogel point of CG and CG/PDA@Ag (CPA2) was around 100% (**Figure** **3**A). Then, as depicted in Figure 3B, the dynamic frequency sweep results indicated that G′ (≈ 1021 Pa) was always larger than the *G*″ (≈312 Pa) under progressively enhanced frequencies from 0.1 to 10 Hz, indicating dominant elastic behavior of CPA2 hydrogel.^[^ ^32^ ^]^ Subsequently, a dynamic step–strain rheological experiment was carried out to quantitatively verify the self‐healing capability of designed hydrogels (Figure 3C). A rheological recovery test was first carried out by measuring the storage modulus (*G*′) and the loss modulus (*G*″) of CG/PDA@Ag hydrogel. The small strain was chosen at 1%, and the large strain was fixed at 400% (over four times the critical points). According to Figure 3C, the value of *G*′ was lower than *G*″ when a high strain (400%) was applied, meaning that the collapse of hydrogel occurred. Once the applied dynamic strain returned to 1%, the value of *G*′ sharply enhanced and instantaneously became greater than the *G*″, indicating that the sample had returned to the hydrogel state. Moreover, both *G*′ and *G*″ could be fully restored to their original values without delay. Of note, this phase transition can be repeated even after three alternately cycles, demonstrating the prominent self‐healing ability of CG/PDA@Ag hydrogel.^[^ ^33^ ^]^ Further, the viscosity of the CG/PDA@Ag hydrogel was investigated (Figure 3D). We found that the viscosity of the CG/PDA@Ag hydrogel decreased when the shear rate enhanced from 0.1 to 10 s^−1^, suggesting the injectability of the hydrogel.^[^ ^34^ ^]^

Next, we used experimental manipulations to verify the feasibility of these rheological data. As displayed in Figure 3E, the CPA2 sample can be continuously injected into the petri dish with a 5 mL syringe, while the formed “WMU” font can be maintained in a hydrogel state, confirming the injectability of the hydrogel. Moreover, the CPA2 with piece or cylinder shapes was physically cut into two halves to verify the self‐healing ability of the hydrogel. As expected, after contacting and placing at ambient conditions for 2 h, the two cut halves automatically rejoined without any external intervention and recombined into a new one without a significant fracture line. Besides, no apparent difference between the healing hydrogel and original hydrogel was observed (Figure 3F). We hypothesized that the resultant CPA2 hydrogel achieving efficient self‐healing ability was attributed to the noncovalent interactions (hydrogen bonds, electrostatic forces, and *π*–*π* stacking) mediated by the functional groups of polysaccharide chains and the catechol groups of the PDA@Ag, which can be dynamically disassociated and associated (Figure 3G).^[^ ^30^, ^31^ ^]^

### 2.3 In Vitro Photothermal Performance of CG/PDA@Ag Hydrogel

As described above, PDA@Ag nanoparticles were incorporated into CG hydrogel to avoid the loss of photothermal performance caused by the aggregation of nanomaterials. To validate this hypothesis, the photothermal conversion efficiency (*η*) of PDA@Ag and CG/PDA@Ag at 808 nm was first assessed. At the same concentration of PDA@Ag nanoparticles, the *η* of PDA@Ag and CG/PDA@Ag was calculated to be 36.1% and 38.2% (Figure S9, Supporting Information), which was much higher than other reported systems,^[^ ^35^ ^]^ indicating our speculation that preventing aggregation and promoting dispersion of PDA@Ag nanoparticles facilitated the improvement of the photothermal performance of the preparation system. Thus, CG/PDA@Ag hydrogel was selected as a model sample to conduct the following antibacterial tests.

**Figure 4:**
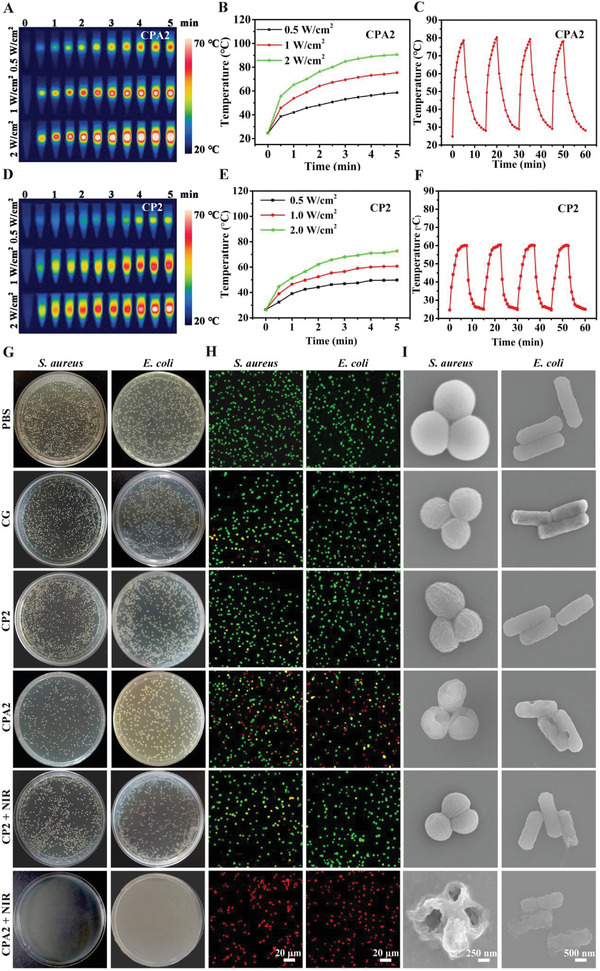
In vitro photothermal and antibacterial performance. Photothermal properties of CG/PDA@Ag (CPA2) hydrogel: A) infrared thermal images and B) photothermal temperature rise curves under 808 nm NIR laser irradiation with various laser power densities (0.5, 1.0, and 2.0 W cm^−2^); C) photothermal stability under repeated laser on‐off of 808 nm NIR irradiation at a power density of 1.0 W cm^−2^. Photothermal properties of CG/PDA (CP2) hydrogel: D) infrared thermal images and E) photothermal temperature rise curves under 808 nm NIR laser irradiation with various laser power densities (0.5, 1.0, and 2.0 W cm^−2^); F) photothermal stability under repeated laser on‐off of 808 nm NIR irradiation at a power density of 1.0 W cm^−2^. Antibacterial activity of samples: G) colonization, H) confocal fluorescence, and I) SEM photographs of *S. aureus* and *E. coli* after treatments with PBS, CG, CP2, CPA2, CP2 + NIR, and CPA2 + NIR.

The photothermal ability of the CG/PDA@Ag (CPA2) hydrogel was first investigated under different power. Specifically, as shown in **Figure** **4**A,B, the temperature of the CPA2 hydrogel enhanced from 25 to 50 °C in 3 min and achieved 58.6 °C in 5 min under the irradiation of 808 nm NIR laser (0.5 W cm^−2^). As the laser power enhanced, the temperature of CPA2 hydrogel rose to 75.4 °C (1.0 W cm^−2^) and 90.6 (2.0 W cm^−2^) after 5 min of 808 nm NIR irradiation. Moreover, CG/PDA@Ag hydrogel still maintained extraordinary photothermal stability even after multiple cycles of on‐off NIR irradiation as there was no significant decrease in the maximum heating temperature (Figure 4C). Compared to the CPA2 hydrogel, the pure PDA‐modified hydrogel (CP2) exhibited a weaker photothermal heating capacity (Figure 4D–F). For example, the central temperature of the CPA2 gel was 70.1 °C at 1 W 808 nm irradiation for 3 min, while the CP2 hydrogel was only 54.5 °C. These results demonstrated that the designed CG/PDA@Ag hydrogel exhibited an outstanding photothermal performance, which was potentially a photothermal platform for antibacterial therapy.

### 2.4 In Vitro Photothermal Antibacterial Property

The outstanding photothermal conversion performance of as‐prepared CG/PDA@Ag hydrogel inspires us to evaluate its antibacterial ability. Herein, the in vitro antibacterial activity of the CG/PDA@Ag hydrogel was explored against two bacterial strains, Gram‐negative *E. coli* and Gram‐positive *S. aureus*, which were responsible for most infectious diseases.^[^ ^36^ ^]^ As presented in Figure 4G and Figures S10 and S11 (Supporting Information), after contact with the pure CG hydrogel at 37 °C, the CG group displayed a 75.2% survival ratio for *E. coli* and 78.5% survival ratio for *S. aureus*, respectively. This phenomenon was because the CG hydrogel possessed abundant functional groups such as positively charged quaternary ammonium salt groups that can interact with the negatively charged bacteria cell membrane, resulting in pathogen capture and inactivation.^[^ ^37^ ^]^ Besides, Ag‐containing nanoparticles also had a certain antibacterial effect, and the sustained release of Ag ions contributed to the long‐term antimicrobial efficacy of the prepared CG/PDA@Ag system (Figure S12, Supporting Information).^[^ ^3^ ^]^ For example, compared to CP2 hydrogel containing PDA without Ag modification, CPA2 hydrogel had further enhanced bactericidal ability, which can kill 46.1% of *E. coli* and 48.9% of *S. aureus*. Next, after NIR irradiation, the temperature of CPA2‐incorporated bacterial suspension increased significantly, from 37 to 67.3 °C (Figure S13, Supporting Information). Notably, almost all *E. coli* (99.9%) and *S. aureus* (99.8%) were inactivated by the CPA2 hydrogel with the assistance of 808 nm NIR laser (1 W cm^−2^, 3 min). In contrast, under the same time and power of NIR irradiation, the temperature rise of CP2‐contained bacterial solution was minimal (from 37 to 49.1 °C), killing only 35.3% of *E. coli* and 39.8% of *S. aureus*. These results suggested that, under the synergistic effect of CG hydrogel matrix (bacteria capture) and PDA@Ag nanoparticles (Ag ion release and photothermal sterilization), the designed CG/PDA@Ag platform exhibited robust antibacterial activity.

To further explore the antibacterial effect of the CG/PDA@Ag (CPA2) hydrogel, a live/dead cell assay by SYTO9/PI was performed. All bacteria were stained by SYTO9 and emitted green fluorescence, whereas the dead bacteria were labeled by PI and emitted red fluorescence.^[^ ^38^ ^]^ It can be seen in Figure 4H and Figures S14 and S15 (Supporting Information) that both *S. aureus* and *E. coli* treated by PBS presented noticeable green fluorescence, and only a few bacteria were labeled with red fluorescence. In contrast, some red fluorescence appeared in the CG hydrogel treatment group. Besides, the red color gradually became more after PDA@Ag doped CG hydrogel, and the CPA2 + NIR treatment group displayed the strongest red fluorescence, reflecting the synergistic eradication effect on both *S. aureus* and *E. coli*. Interestingly, the red fluorescence of the PDA‐loaded hydrogel (CP2) did not change significantly before and after light exposure. This phenomenon was because the photothermal property of CP2 hydrogel was weak, which brought about a temperature increase (49.1 °C, Figure S13, Supporting Information) that was not sufficient to kill bacteria. It was worth mentioning that the fluorescence‐based test and plate counting assay results had the same antibacterial trend, further verifying the great potential of CG/PDA@Ag hydrogel for effectively antibacterial therapy through the cooperation between hydrogel and PTT.

In addition to the plate counting approach and live/dead staining assay, the morphology changes of *S. aureus* and *E. coli* after different treatments were explored using SEM to characterize the bactericidal effect. As illustrated in Figure 4I, both *S. aureus* and *E. coli* treated by PBS presented intact and smooth cell membrane, manifesting that the damage to bacteria by PBS was negligible. In contrast, some wrinkles were appeared in the CG hydrogel treated group, indicating that the hydrogel possessed a particular antibacterial ability. Nevertheless, some well‐shaped bacteria were still observed in the CG hydrogel group, suggesting that the antibacterial performance was insufficient to kill all bacteria. After Ag@PDA doping, the morphology of bacteria was further disrupted, and some shrinkage and pores started to appear, indicating that the antibacterial ability of the CG system was further enhanced. However, with the help of PDA and NIR, the morphology of the bacteria hardly changed compared with the pure CG group, suggesting that the PDA itself and the heat‐induced by PDA were not effective in enhancing the antibacterial activity of the resulting hydrogel. More importantly, the bacterial cell membrane showed much more severe damage, and the bacteria was distorted or even ruptured when CPA2 hydrogel was applied in the presence of NIR. These results confirmed that the antibacterial platform (CPA2 + NIR) possessed the most substantial bactericidal effect, inducing the destruction of the bacterial cell membrane.^[^ ^37^ ^]^

### 2.5 In Vitro Hemolysis and Cytotoxicity Assay

Biocompatibility is crucial for the in vivo antibacterial application of a hydrogel.^[^ ^33^, ^39^ ^]^ Hemocompatibility and cytocompatibility experiments were conducted to evaluate the biocompatibility of the CG‐based hydrogels (CG, CP2, and CPA2) initially. The in vitro hemocompatibility was first tested. The supernatant of red blood cells treated by Triton displayed bright red, while the supernatants of hydrogel and PBS groups were colorless and transparent (Figure S16, Supporting Information). Accordingly, as presented in Figure S17 (Supporting Information), the optical density (OD) values of PBS and hydrogel groups at 545 nm were much lower than the Triton treated group; the hemolysis ratios of CG, CP2, and CPA2 hydrogels were <5%, which was considered a safe level of hemostatic materials. These data reflected the negligible hemolysis of designed hydrogels on red blood cells. Subsequently, the cytotoxicity of the hydrogels to L929 cells was assessed by the CCK‐8 assay. In contrast to the control group, the hydrogels presented no significant cytotoxicity to L929 fibroblast cells after 1, 3, and 5 d of coculture (Figure S18, Supporting Information). The cytocompatibility of the as‐fabricated hydrogels was further explored by the live/dead cell staining technique. As shown from Figure S19 (Supporting Information), similar to the control group, no apparent red fluorescence was observed when L929 cells were coincubated with hydrogels. Simultaneously, the hydrogel treatments did not affect cell growth and proliferation (Figure S20, Supporting Information), suggesting that the hydrogels were nontoxic.^[^ ^40^ ^]^ Taken together, the designed CG‐based hydrogels with outstanding biocompatibility were suitable for anti‐infective therapy in vivo.

### 2.6 In Vivo Antibacterial Assay

**Figure 5:**
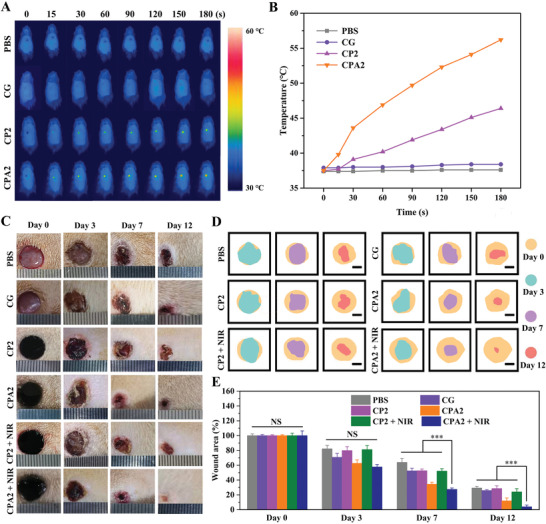
In vivo assessment of the NIR light‐induced antibacterial properties of CG/PDA@Ag hydrogel. A) Infrared thermal images and B) corresponding temperature elevation at the wound sites of rats in the PBS and hydrogels groups under 808 nm NIR irradiation (1.0 W cm^−2^) at determined times. C) Photographs of *S. aureus*‐infected wounds treated with different samples from day 0 to day 12. D) Schematic images of wound contraction with different treatments from day 0 to day 12. E) Wound area for each group (*n* = 3, **P < 0.01, ***P < 0.001, and NS means not significant).

The in vivo antibacterial potential of the developed CG/PDA@Ag hydrogel was evaluated using a rat bacteria‐infected model. In vivo photothermal performance was first assessed using an infrared camera after the hydrogels were injected onto the back skin wounds of the rat. As shown in **Figure** **5**A,B, after 3 min of 808 nm NIR irradiation (1.0 W cm^−2^), the wound region temperature of CG/PDA@Ag (CPA2) hydrogel was raised to 56.2 °C, while the local temperature of the hydrogels without PDA@Ag nanoparticles was only enhanced to 35.0 °C (CG) and 46.4 °C (CP2) under the same conditions. After different treatments, bacteria at the wound sites were detected by the agar plate approach to evaluate the bactericidal effect (Figures S21 and S22, Supporting Information). Clearly, a large number of bacteria on the agar plates of the control group were observed, and the number of bacteria started to decrease when the hydrogel and NIR were treatments. As expected, CG/PDA@Ag + NIR group displayed almost no bacteria on the agar plate, which was in line with the in vitro results. These results offered intuitive proof that the CG/PDA@Ag could be a robust platform for antibacterial therapy.

Moreover, the wound healing process of rats treated by different groups (PBS, CG, CP2, CPA2, CP2 + NIR, and CPA2 + NIR) was monitored photographically (Figure 5C). The corresponding schematic images of wound contraction and calculation of wound contraction were presented in Figure 5D,E, respectively. For all tested groups, the wound size gradually reduced over time. On day 12, the wound treated with CPA2 + NIR possessed the smallest open wound (3.9%) and became smooth with some new epidermal and dermal tissues, whereas 23.9%, 11.8%, 28.5%, 25.8%, and 29.3% of the wound remained open with apparent uneven scar tissues in the CP2 + NIR, CPA2, CP2, CG, and PBS groups, respectively. These results demonstrated that the CPA2 + NIR group possessed the best wound healing efficacy compared to the other groups. The NIR‐assisted ablation effect of PDA@Ag nanoparticles and the inherent antibacterial ability of hydrogel synergistically increased the antibacterial activity of the designed platform, which can efficiently kill pathogens and accelerate the process of wound healing. In addition, some functions provided by the hydrogel at the wound interface like hemostasis (Figures S23 and S24, Supporting Information), exudate adsorption, serving as a barrier to microorganisms, gaseous exchange, and acting as a scaffold for cells, may also be beneficial for promoting wound healing.^[^ ^21^, ^41^ ^]^

**Figure 6:**
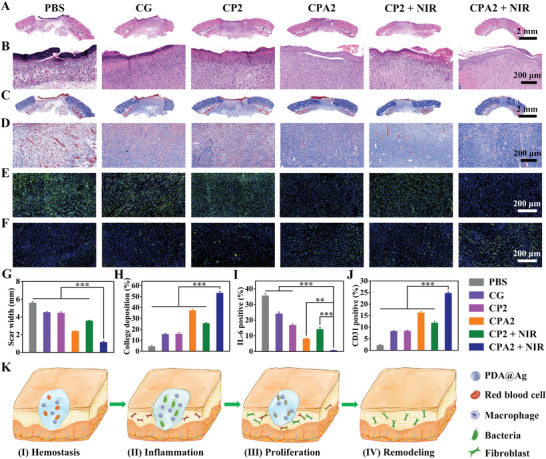
Histological changes in healed skin tissues after treatment with different formulas. A,B) H&E staining, C,D) Masson's trichrome staining, E) IL‐6 staining, and F) CD31 staining of the skin tissues from the wound edges. G–J) Quantitative analysis of stained images acquired from different groups: scar width, college deposition, IL‐6, and CD31 (*n* = 3, **P < 0.01, and ***P < 0.001), respectively. K) Proposed mechanism of the designed CG/PDA@Ag hydrogel for accelerating wound healing.

Furthermore, to verify the above healing results, the detail of the wound healing was assessed by the histological changes in skin tissues. After treatments, rats were sacrificed at a pre‐set time, and tissues from the PBS, CG, CP2, CPA2, CP2 + NIR, and CPA2 + NIR groups were collected. These skin tissues were fixed with formalin, embedded with paraffin, sliced with slicer, and stained with hematoxylin and eosin (H&E) (**Figure** **6**A,B), Masson (Figure 6C,D), IL‐6 (Figure 6E), and CD31 (Figure 6F). The H&E staining results proved that on day 12, the scar width of the CPA2 + NIR treated group (1.15 mm) was the most minor compared to the PBS (5.59 mm), CG (4.52 mm), CPA2 (4.46 mm), CPA2 (2.40 mm), and CP2 + NIR (3.58 mm) groups; simultaneously, higher magnification H&E images revealed that many inflammation signals (blood cells and neutrophils) appeared in the skin tissue of control group and there was some reduction in inflammatory cells after CG and CPA2 hydrogel treatment (Figure 6A,B,G). In contrast, for CPA2 + NIR group, no evident inflammation occurred, and the regenerated dermis tissue with appendages like hair follicles was detected. Moreover, more collagen fibers appeared in the CPA2 + NIR group than in other groups (Figure 6C,D,H). Besides, immunofluorescence staining of DAPI and IL‐6 revealed that the expression of inflammation marker of IL‐6 in the wound area was significantly lower in the CPA2 + NIR group than in the other five groups (Figure 6E,I).^[^ ^42^ ^]^ Additionally, the NIR‐assisted CPA2 hydrogel presented the highest expression of CD31 than the other tested groups, suggesting the best pro‐vascularization ability of the CPA2 + NIR group (Figure 6F,J).^[^ ^43^ ^]^ Importantly, when compared to the CP2 hydrogel incorporating pure PDA nanoparticles without Ag deposition, the CPA2 hydrogel containing Ag‐modified PDA nanoparticles exhibited a higher photothermal heating capacity (>50 ℃) with the help of NIR and can effectively kill bacteria, thus accelerating the transition from the inflammatory to the repair phase of the wound. In sum, the wounds treated by the CPA2 + NIR group showed good hemostasis capability, low inflammation, high collagen fiber content, thick granulation tissue, remarkable hair follicle regeneration, and rapid wound healing rate, which can promote the healing of a wound by accelerating phase transition of the wound from the hemostasis to inflammation, proliferation, and remodeling (Figure 6K). More importantly, after realizing these functions, the CPA2 dressing can be wholly absorbed by the organism without additional secondary removal, ensuring ease of use in practice (Figure S25, Supporting Information). These results confirm that CG/PDA@Ag hydrogel is a promising platform for in vivo antibacterial therapy and wound healing.

Finally, the biosafety of our designed hydrogel dressings was investigated in vivo to confirm their potential for future application of bacteria inhibition. As shown in Figure S26A (Supporting Information), the weights of rats gradually grew in both the control and experimental groups, proving that these treatments had no systemic harmful effects on rats. Moreover, routine blood tests and blood biochemical analysis showed no significant fluctuations in all parameters in the treated rats compared to the control group (Figure S26B–O, Supporting Information), demonstrating that kidney and liver functions were not affected. In addition, no significant pathological abnormalities or injuries were found in the major organs of the rats in each group (Figure S26P, Supporting Information). These results consistently verified the excellent biological security of PTT using PDA@Ag loaded CG hydrogel, exhibiting the great potential of this approach in clinical translation.

## 3 Conclusion

In summary, an innovative antibacterial platform (CG/PDA@Ag) was rationally developed and successfully constructed. First, PDA@Ag nano‐PTAs were fabricated by growing Ag nanocrystals on the surface of PDA nanoparticles, and subsequently, they were uniformly encapsulated in a CG hydrogel matrix by a simply one‐pot bending method. Mechanistically, thanks to the high photothermal conversion efficiency as well as inherent bacterial capture/killing ability of the hydrogel matrix, an environmentally friendly, facile, broad‐spectrum, and highly efficient antibacterial platform was achieved, not only ensuring high‐efficiency bacteria‐killing ability toward *E. coli* (99.9%) and *S. aureus* (99.8%) in vitro but also realizing superior bactericidal activity in vivo. For the first time, this work provides new ideas for PDA nano‐photothermal agents to achieve robust antibacterial performance from material modification to application.

## Conflict of Interest

The authors declare no conflict of interest.

## Supporting information

Supporting information

## Data Availability

The data that support the findings of this study are available from the corresponding author upon reasonable request.
