# Supplementary material for: Engineering Robust Ag‐Decorated Polydopamine Nano‐Photothermal Platforms to Combat Bacterial Infection and Prompt Wound Healing
Source: Adv Sci (Weinh). 2022 Feb 22;9(11):2106015. doi: 10.1002/advs.202106015 (PMC9008420; doi:10.1002/advs.202106015)
Supplement: Supplementary file 1 — Supporting information [file ADVS-9-2106015-s001.pdf]

Supporting Information

Engineering robust Ag-decorated polydopamine nano-photothermal platforms to combat bacterial infection and prompt wound healing

Xiaoliang Qi [a, 1], *Yijing Huang* [b, 1], *Shengye You* [c], *Yajing Xiang* [c], *Erya Cai* [c], *Ruiting Mao* [c], *Wenhao Pan* [c], *Xianqin Tong* [c], *Wei Dong* [b, *], *Fangfu Ye* [d, *], *Jianliang Shen* [a, d, e, *]

[a] State Key Laboratory of Ophthalmology, Optometry and Vision Science, School of Ophthalmology and Optometry, School of Biomedical Engineering, Wenzhou Medical University, Wenzhou, Zhejiang 325027, China
[b] School of Chemical Engineering, Nanjing University of Science & Technology, Nanjing, Jiangsu 210094, China
[c] School & Hospital of Stomatology, Wenzhou Medical University, Wenzhou, Zhejiang 325027, China
[d] Wenzhou Institute, University of Chinese Academy of Sciences, Wenzhou, Zhejiang 325000, China
[e] Oujiang Laboratory (Zhejiang Lab for Regenerative Medicine, Vision and Brain Health), Wenzhou, Zhejiang 325001, China

[1] These authors contributed equally to this work.

[*] Corresponding authors. E-mail addresses: weidong@njust.edu.cn (W. Dong), fye@iphy.ac.cn (F. Ye), and shenjl@wiucas.ac.cn (J. Shen).

Materials and instrumentation

Materials

Cationic guar gum (CG) was supplied by Usolf (Shandong, China) and further purified before use. Dopamine hydrochloride, ammonium hydroxide (30 wt% in water), anhydrous ethanol, and sodium hydrate were purchased from Aladdin (Shanghai, China). Silver nitrate was purchased from Alfa Aesar (Shanghai, China). LIVE/DEAD™ BacLight™ Bacterial Viability Kit comprising propidium iodide (PI) and SYTO9 was purchased from Thermo Fisher Scientific (L7012, Waltham, USA). Dulbecco's modified Eagle medium (DMEM), fetal bovine serum (FBS), Calcein-AM/PI Cell Viability Kit, penicillin-streptomycin (P/S), trypsin, Cell Counting Kit-8 (CCK-8), Triton X-100, and phosphate-buffered saline (PBS, pH = 7.4) were purchased from Beyotime Biotechnology (Shanghai, China). All reagents were used without further purification or modification.

Fabrication of PDA nanoparticles

In this work, PDA nanoparticles were synthesized based on our previous reports with slight modifications.[1] Specifically, aqueous ammonia solution (2 mL, 28–30%) was mixed with 90 mL of deionized water and 40 mL of anhydrous ethanol under mild stirring at 25 ℃ for 0.5 h. Then, dopamine hydrochloride (500 mg) was dissolved in 10 mL of deionized water and slowly poured into the above-mixed solution. After that, the solution was continued to stir for 24 h gently. PDA nanoparticles collected by high-speed centrifugation (12,000 rpm) for 8 min were washed five times with deionized water and anhydrous ethanol. Ultimately, the prepared PDA nanoparticles were diluted to 10 mg/mL and stored in the refrigerator (4 °C) for long-term usage.

Fabrication of PDA@Ag nanoparticles

Briefly, 500 mg of $AgNO_3$ was dissolved in deionized water. Ammonia solution was then added to the above solution until the resulting brown precipitate disappeared and the solution became clarified again. Next, PDA nanoparticles (0.3 mL, 10 mg/mL) were added to the solution. The mixed solution was gently stirred at 25 ℃ for 1 h away from light and centrifuged at 12,000 rpm to obtain Ag-decorated PDA (PDA@Ag) nanoparticles.

Fabrication of hydrogels

In detail, 400 mg cationic guar gum (CG) powder was added to deionized water (10 mL) under vigorous stirring at 25 °C for 30 min. Afterward, different volumes of PDA@Ag solution (10 mg/mL) were added to the above solution. The final volume of the reaction solution was made up to 20 mL using deionized water. After vigorous stirring at 25 °C for 2 min, the mixed solution was kept at 25 °C to form a hydrogel. In this work, hydrogels with CG/PDA@Ag weight ratios of 80:0, 80:1, 80:2, and 80:4 were fabricated (designated as pure CG, CPA1, CPA2, and CPA3, respectively). Besides, hydrogels with CG/PDA weight ratios of 80:1, 80:2, and 80:4 were also fabricated (designated as CP1, CP2, and CP3, respectively) to verify the performance of hydrogels incorporated with different nanoparticles.

Structural characterization

The morphology of different samples was observed by a scanning electron microscope (SEM, SU-8010, Hitachi, Japan) and a transmission electron microscope (TEM, JEM-1230, JEOL, Japan). X-ray diffraction (XRD) patterns of PDA and PDA@Ag nanoparticles operated at 30 kV, and 20 mA were acquired with Cu Kα1 (λ = 0.15418 nm) radiation from 5° to 80° using a Bruker AXS D8 Advance diffractometer (Germany). The zeta potentials of nanoparticles were determined by a

ZEN3600 particle size analyzer (Malvern, UK). The ultraviolet–visible–near-infrared (UV–vis–NIR) absorption spectra were obtained by a Cary5000 spectrophotometer (Agilent, USA). The chemical composition of samples was analyzed by a Tensor II Fourier transform infrared (FTIR) spectrometer (Bruker, Germany). Thermogravimetric analysis (TGA) was performed with a thermal gravimetric analyzer (TGA8000, PerkinElmer, USA) at a 10 °C/min heating ramp from 30 ℃ to 600 ℃ under nitrogen flow. The rheological properties of hydrogels were determined by a rheometer (DHR-2, TA, USA), and four different rheological tests were conducted:[2] (1) dynamic strain sweep measurements were first conducted to acquire the linear viscoelastic regions of hydrogels; (2) the self-healing behavior of hydrogels was investigated by determining the storage modulus (G′), and loss modulus (G″) at alternate oscillation strain of 1% and 400%; (3) shear rate sweep tests of hydrogels were carried out and (4) dynamic frequency sweep experiments were carried out within 0.1–10 Hz at a fixed strain of 1% at 25 ℃.[3]

Swelling, water retention, and degradation tests

To determine the swelling behavior, the pre-weighed freeze-dried hydrogel specimens (W_d) were incubated in PBS at 37 °C until the equilibrium state was achieved. During the swollen process, hydrogels were taken out at specified durations, residual liquids on the hydrogel surface were removed using wet filter papers, and the wet weights (W_w) of hydrogels were recorded using an electronic balance (BSA224S-CW, Sartorius, Germany).[4] The swelling ratio was defined as:

$$\textit{Swelling ratio} = (W_w - W_d)/W_d \qquad (1)$$

where W_w is the mass of the swollen hydrogel specimen, and W_d is the mass of the initial specimen.

To evaluate the water retention capacity, freeze-dried hydrogel samples were pre-immersed in PBS to reach swelling equilibrium (W_0). Subsequently, these swelling equilibrium specimens were transferred into a 37 °C oven, and their masses

(W_t) were determined at different time lengths. The water retention of hydrogels can be calculated according to the following equation: [5]

$$\text{Water retention (\%)} = W_t/W_0 \times 100\% \quad (2)$$

where W_t is the mass of the hydrogel specimen at a given time point, and W_0 is the mass of the initial hydrogel.

To assess the degradation characteristics of the designed hydrogel, the sample was first immersed in PBS to reach swelling equilibrium. At a predetermined time, the morphology change and mass change of the hydrogels cultured in PBS were photographed and weighed, respectively.

$$\text{Degradation ratio (\%)} = W_t/W_s \times 100\% \quad (3)$$

where W_t is the mass of the hydrogel specimen at a given time point, and W_s is the mass of the fully swollen hydrogel.

In vitro Ag release

First, the CPA2 hydrogel was weighed and then immersed in 40 mL of PBS solution. At each preset time, 4 mL of PBS solution was collected and supplemented with 4 mL of fresh PBS buffer. Subsequently, the Optima 8000 inductively coupled plasma optical emission spectrometry (PerkinElmer, USA) was used to quantify the amount of released silver. The time points for measuring released Ag ions were 6, 12, 24, 48, 72, 96,120, and 144 h.

Measurements of photothermal performance

The photothermal performance of the prepared samples (nanoparticles and hydrogels) was evaluated using an 808 nm NIR laser. Briefly, all samples were added into 1.5 mL Eppendorf tubes and irradiated with an 808 nm NIR laser for a pre-set

time at different power settings (0.5, 1.0, and 2.0 W/cm^2). During irradiation, the temperatures of the samples were captured by an E4 FLIR infrared thermometer (USA). Besides, the photothermal stability of samples during four circles of heating-cooling processes was explored.

***In vitro* antibacterial assay**

The antibacterial activity of CG-based hydrogels against *Staphylococcus aureus* (*S. aureus*, ATCC 29213) and *Escherichia coli* (*E. coli*, ATCC 25922) was investigated *in vitro* using the spread plate method. Typically, hydrogel pieces with a diameter of 8 mm were first pre-soaked in PBS until reaching absorption equilibrium. Subsequently, they were sterilized with a UV lamp for 30 min and then immersed into bacteria suspension (1 mL, 1.0 × 10^8 CFU/mL). After co-incubating at 37 °C for 4 h, these samples were treated with or without NIR irradiation (808 nm, 1 W/cm^2) for 3 min. Afterward, 100 μL of the bacterial solution was taken out from each group and diluted. Then, 100 μL of the diluted bacterial suspension was uniformly spread onto the fresh Luria Bertani broth agar plates. After cultivation overnight, the bacterial colonies on the agar plates were photographed and counted using a J3 colony counter (Tenlin, China). The bacteria suspension treated by PBS with or without NIR irradiation was considered the control group.

Besides, we studied the antibacterial properties of hydrogels by a bacterial live/dead staining assay. Briefly, after different treatments, the bacteria cells were co-stained by PI and SYTO9 for 20 min in the dark, followed by washing thrice with PBS. According to the manufacturer's instructions, all bacteria were labeled by SYTO9 and appeared green fluorescence, while dead bacteria were stained by PI and revealed red fluorescence. Finally, fluorescence images were captured using confocal laser scanning microscopy (CLSM, A1 imaging system, Nikon, Japan).

In addition, SEM imaging was also performed to visualize the bacteria morphological changes after the antibacterial experiments. Specifically, after treatments, all collected bacteria were washed with PBS and then fixed with 2.5% glutaraldehyde solution at 4 °C for 40 min. After fixation, these specimens were serially dehydrated by graded ethanol solutions (20–100%). Next, the dried bacteria were sputter-coated with platinum to increase conductivity, and their morphologies

were observed by SEM.

In vitro cytotoxicity assessment

CCK-8 assays were conducted to estimate the cytotoxicity of designed hydrogels. Before cytotoxicity experiments, the designed hydrogels were sterilized using UV light and then added into a DMEM medium with 10% FBS and 1% P/S for 1, 3, and 5 days to obtain the leaching liquid. At the same time, L929 cells were seeded into a 96-well plate at a density of 1×10^4 cells per well. The old cell culture medium in each well was discarded after incubation overnight. Subsequently, the hydrogel extract liquid (100 μL) was added, and the L929 cells were cultured for 4 h. Next, the hydrogel extract liquid was replaced with an equivalent fresh cell culture medium containing 10% CCK-8 for another 4 h of incubation. Ultimately, the solution absorbance at the wavelength of 450 nm was detected employing a microplate reader (1530, Thermo Fisher, Finland). The L929 cells incubated without hydrogel were served as a blank control. Cell viability was defined as:

$$Cell\ viability\ (\%) = A_s/A_c \times 100\% \qquad (4)$$

where A_s and A_c represent the absorbance values of the hydrogel sample and the control sample at 450 nm, respectively.

In addition, the cell viability of the prepared hydrogels (CG, CP2, and CPA2) was investigated using a Calcein-AM/PI Cell Viability Kit according to the manufacturer’s protocol. Briefly, the L929 cells were cultured and seeded as before. After being treated with different groups, the cells in each group were co-stained with Calcein-AM and PI dyes for 20 min. Next, dyed cells were captured using CLSM. The dead cells possessing compromised plasma membranes displayed red fluorescence, whereas viable cells possessing esterase activity showed green fluorescence.[6]

In vitro blood compatibility

The *in vitro* hemolytic activity of the developed hydrogels (CG, CP2, and CPA2) was investigated using rat's red blood cells. Typically, the red blood cells were acquired by centrifuging the fresh rat blood with 2% ascorbic acid for 10 min, followed by rinsing with PBS three times. 1 mL of diluted erythrocytes was added into a tube. Subsequently, hydrogel samples (100 mg) were introduced into these tubes. Furthermore, 100 mg of 0.1% Triton X-100 and PBS were added as positive and negative controls, respectively. After incubated at 37 °C for 3 h, these erythrocyte suspensions were centrifuged at 2500 rpm/min for 5 min. Finally, the absorbance of supernatants at 545 nm was detected by a UV–vis–NIR spectrometer.

$$Hemolysis\ ratio\ (\%) = \frac{(A_h - A_p)}{(A_t - A_p)} \times 100\% \qquad (5)$$

where A_t, A_p, and A_h represent the supernatant fraction absorbance of Triton, PBS, and hydrogel, respectively.

In vivo photothermal performance

All animal experiments were reviewed and approved by the Institutional Animal Care and Use Committee of Wenzhou Medical University (approved number Wydw7019-0134). Male Sprague-Dawley rats were supplied by Vital River Laboratory Animal Technology Co., Ltd (Beijing, China). Rats were randomly and equally divided into six groups (three rats per group): PBS, CG, CP2, CPA2, CP2 + NIR, and CPA2 + NIR. An 8 mm circular hole was punched on the back of each rat and then covered by hydrogels. The wound sites covering CP2 and CPA2 hydrogels were exposed to an 808 nm NIR laser irradiation (1 W/cm^2) for 3 min. The corresponding thermal images were recorded by a thermal infrared camera.

***In vivo* hemostatic assay**

Rats were randomly divided into four groups (three rats per group): control, gauze, CG, CP2, and CPA2. Briefly, fifty percent length of the rat tail was cut off with a surgical scissor. After 15 s of bleeding, the wound was immediately covered with samples, and the treated rat was placed on a pre-weighed dry filter paper. The wound without treatment served as a control group.

***In vivo* degradation assay**

The *in vivo* degradation property of the prepared hydrogel was investigated by a rat subcutaneous transplantation model. Briefly, sterilized sheet hydrogel (CPA2, diameter = 8 mm) was transplanted subcutaneously to the back of the rat. The morphological and mass changes of the subcutaneously implanted hydrogel were photographed and weighed at predetermined times. The degradation rate was calculated by Equation (3) described above.

***In vivo* wound healing assay**

To establish a bacteria-infected rat wound model, four full-thickness round wounds (diameter = 8 mm) were created on the dorsum of each rat. Subsequently, 10 μL of *S. aureus* (1.0×10^7 CFU/mL) was immediately dripped onto the round wounds and evenly smeared over the wound surface. Subsequently, the wounds in each rat were treated with different samples: 90 μL of PBS, CG hydrogel, CP2 hydrogel, CPA2 hydrogel, CP2 hydrogel + NIR (1 W/cm^2, 3 min), and CPA2 hydrogel + NIR (1 W/cm^2, 3 min). A medical tape was employed to immobilize the hydrogels on the wounds. At the predetermined time intervals (days 0, 3, 7, and 12), photographs of the wounds were obtained using a digital camera. Moreover, the bodyweight of each rat was also measured daily using an electronic balance. Skin tissue samples were harvested on days 3, 7, and 12 for hematoxylin and eosin (H&E), Masson's trichrome,

and immunohistochemical (IL-6 and CD31) analyses.[7] Besides, on day 1, wound exudate was collected, diluted, and spread on agar plates.

In vivo biosafety assay

After the *in vivo* antibacterial and wound healing experiments, the blood from rats was collected for blood biochemistry analysis and blood routine examinations. Simultaneously, the major organs (heart, liver, spleen, kidney, and lung) of rats were harvested and stained with H&E for histological evaluation.

Statistical analysis

All experiments were conducted at least three times unless otherwise noted. The statistical analysis was performed using OriginPro 8.0, followed by a Student's t-test and one-way analysis of variance (ANOVA). $^*P < 0.05$ was considered statistically significant. $^{**}P < 0.01$ and $^{***}P < 0.001$ were considered highly significant.

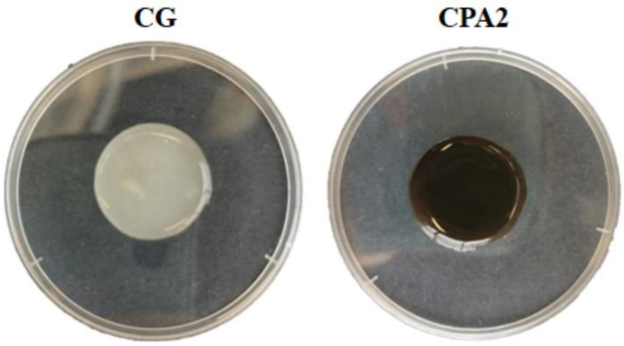

Figure S1. Digital images of CG hydrogel (white) and CPA2 hydrogel (black).

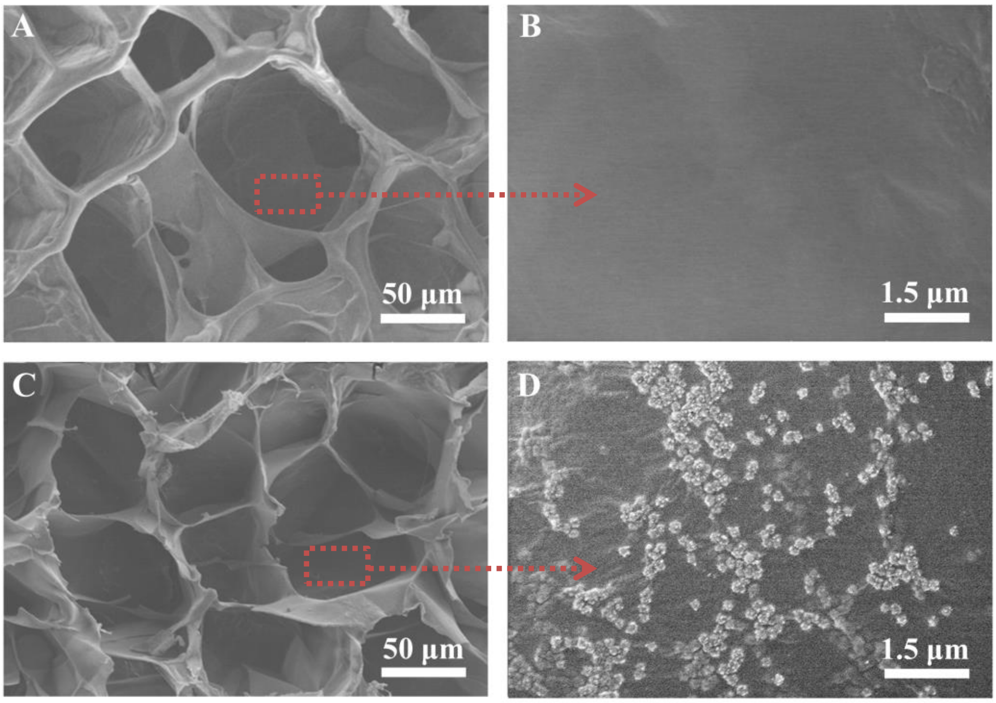

Figure S2. Representative cross-sectional SEM images of CG and CPA2 hydrogels. (A) SEM image of CG hydrogel (scale bar = 50 µm). (B) Enlarged view of the view in (A) (scale bar = 1.5 µm). (C) SEM image of CPA2 hydrogel (scale bar = 50 µm). (D) Enlarged view of the view in (C) (scale bar = 1.5 µm).

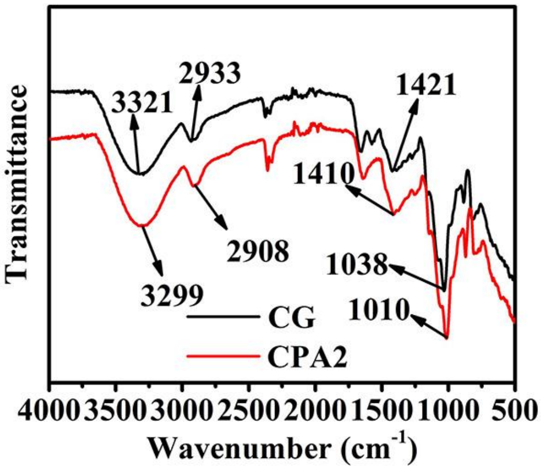

Figure S3. FTIR spectra of CG and CPA2 hydrogels.

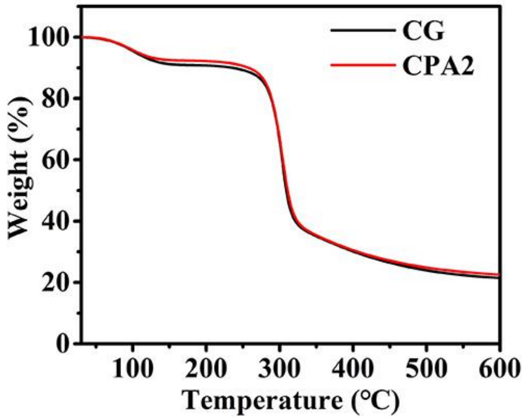

Figure S4. TGA results of CG and CPA2 hydrogels.

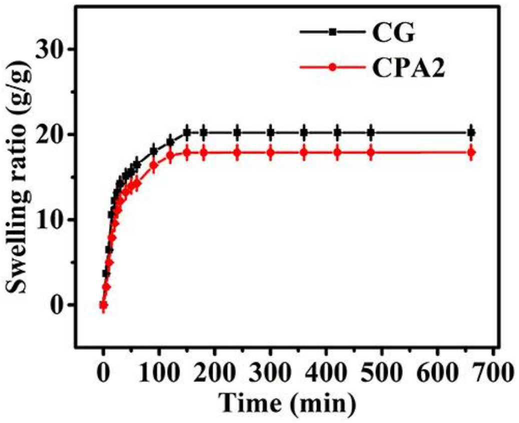

Figure S5. Swelling ratios of CG and CPA2 hydrogels (n = 3).

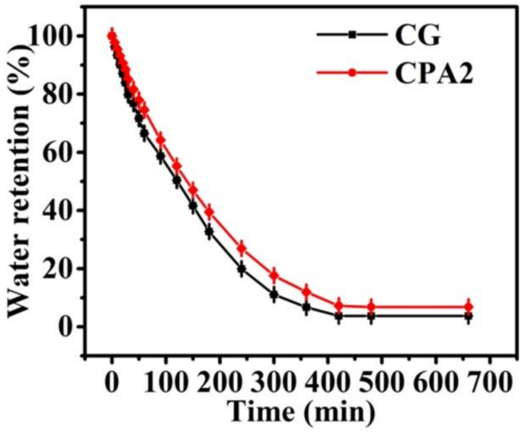

Figure S6. Water retention capacity of CG and CPA2 hydrogels (n = 3).

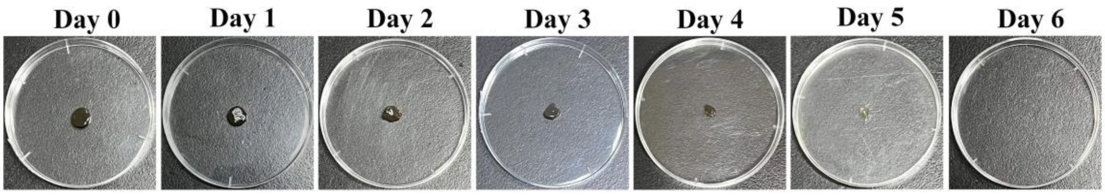

Figure S7. Optical images of the *in vitro* degradation process of CPA2 hydrogel in PBS at 37 ℃.

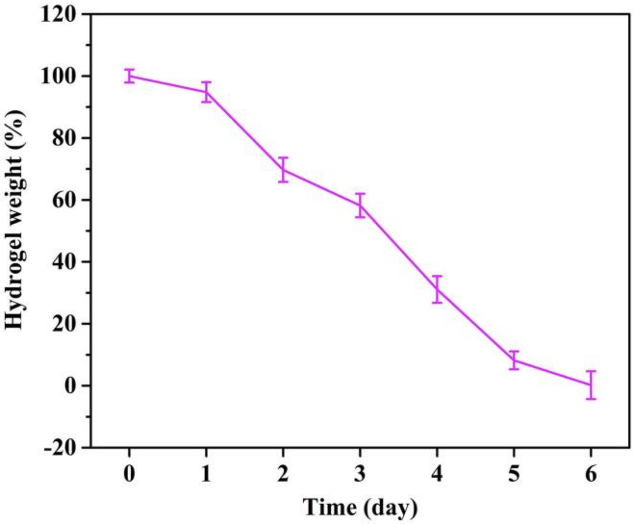

Figure S8. Degradation profile of the *in vitro* degradation process of CPA2 hydrogel in PBS at 37 ℃ (n = 3).

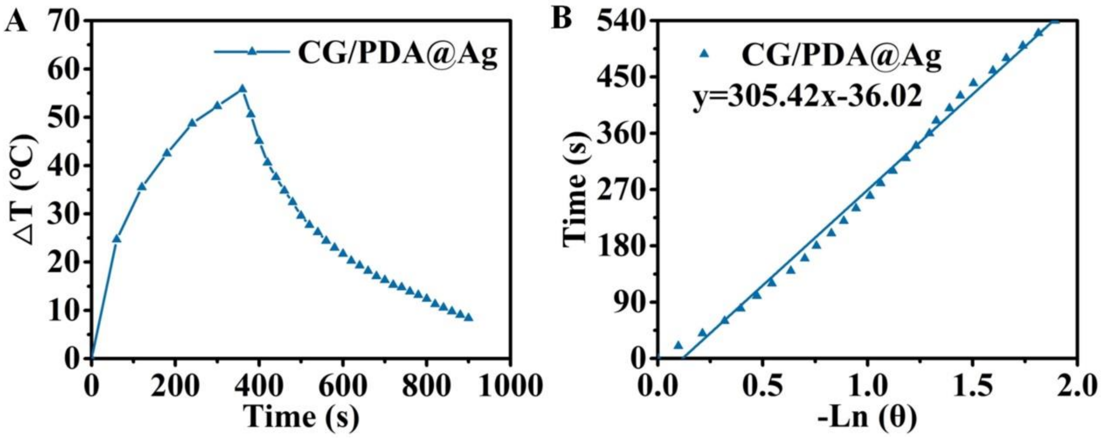

Figure S9. Photothermal effects (A) and plot fitting of cooling time versus the negative natural logarithm of the driving force temperature during the cooling phase (B) of CG/PDA@Ag hydrogel.

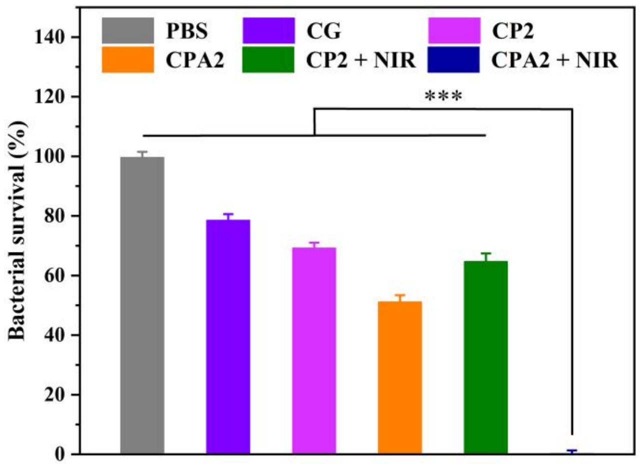

Figure S10. Antibacterial rates of different samples for *S. aureus* (n = 3 and ***P < 0.001).

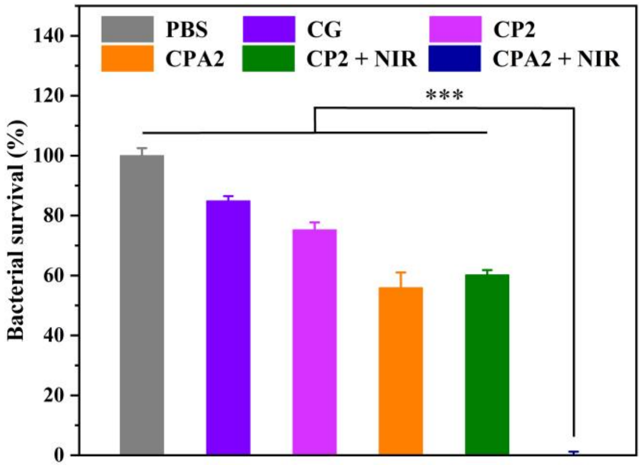

Figure S11. Antibacterial rates of different samples for *E. coli* (n = 3 and ***P < 0.001).

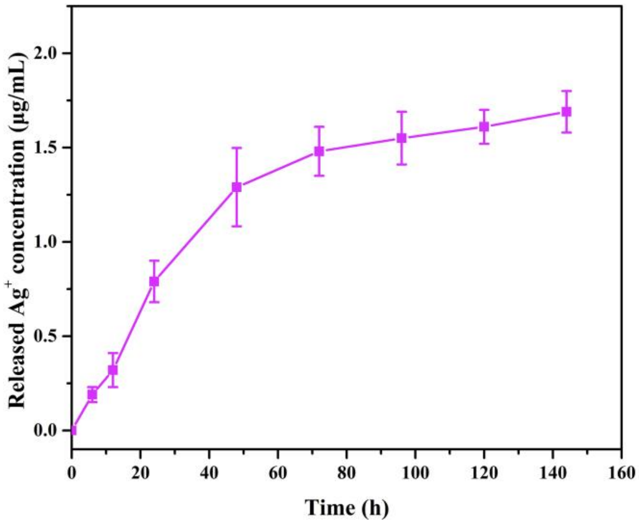

Figure S12. Ag^+ release profile of CG/PDA@Ag hydrogel (n = 3).

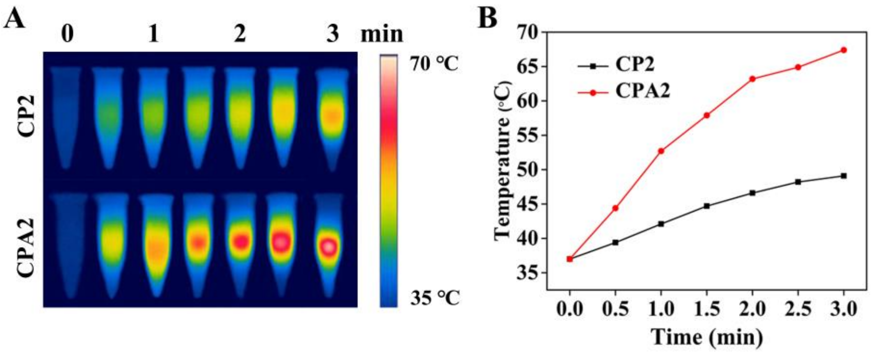

Figure S13. Infrared thermal images (A) and photothermal temperature rise (B) of hydrogel-incorporated bacteria solution under 808 nm NIR laser irradiation (1.0 W/cm^2).

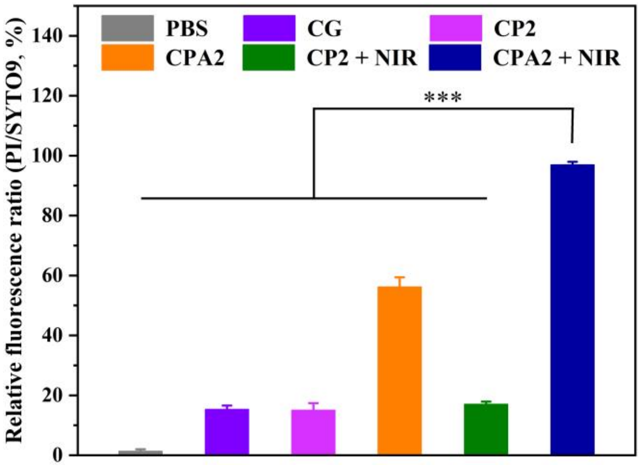

Figure S14. Relative fluorescence ratios of different samples for *S. aureus* (n = 3 and ***P < 0.001).

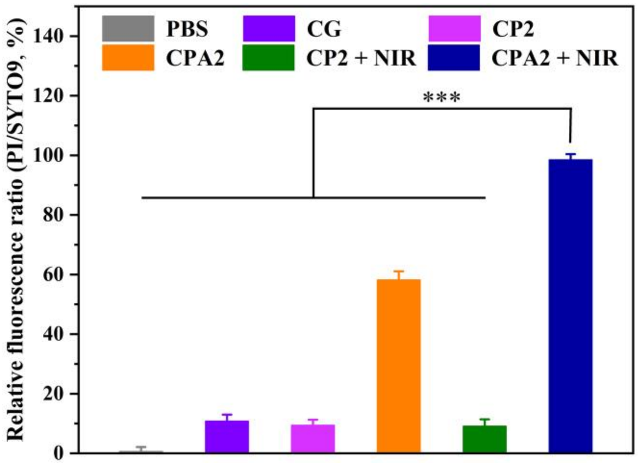

Figure S15. Relative fluorescence ratios of different samples for *E. coli* (n = 3 and ***P < 0.001).

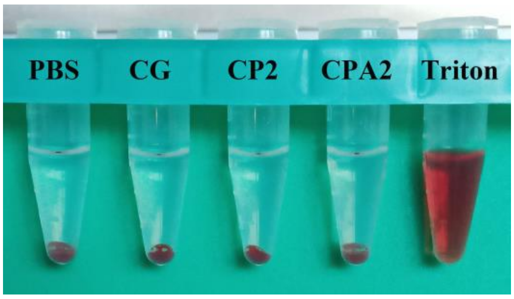

Figure S16. Photographs from hemolysis test on PBS, CG, CP2, CPA2, and Triton (n = 3).

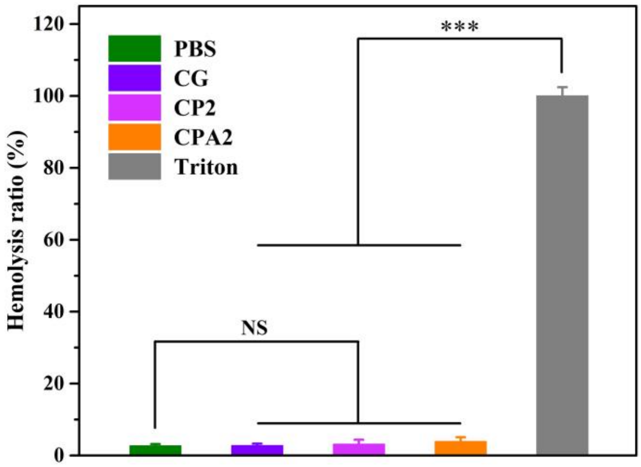

Figure S17. Hemolysis ratios of CG, CP2, and CPA2 hydrogels (n = 3). *** means $P < 0.001$ and NS means not significant.

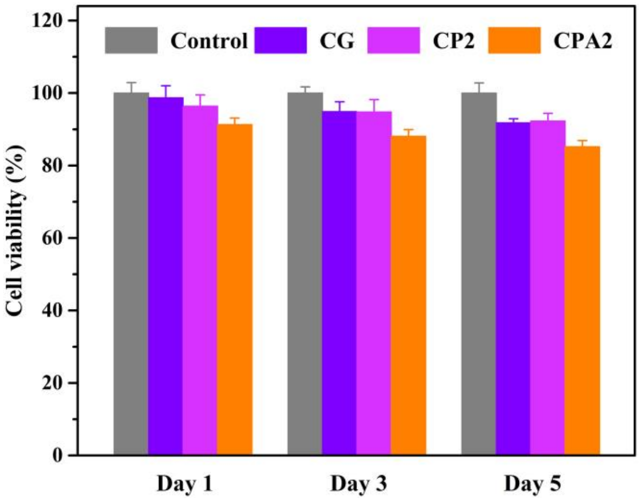

Figure S18. *In vitro* cytotoxicity to L929 cells measured by CCK-8 assay after exposure to the negative control (PBS) and hydrogel samples (CG, CP2, and CPA2) for 1 day, 3 days, and 5 days (n = 3).

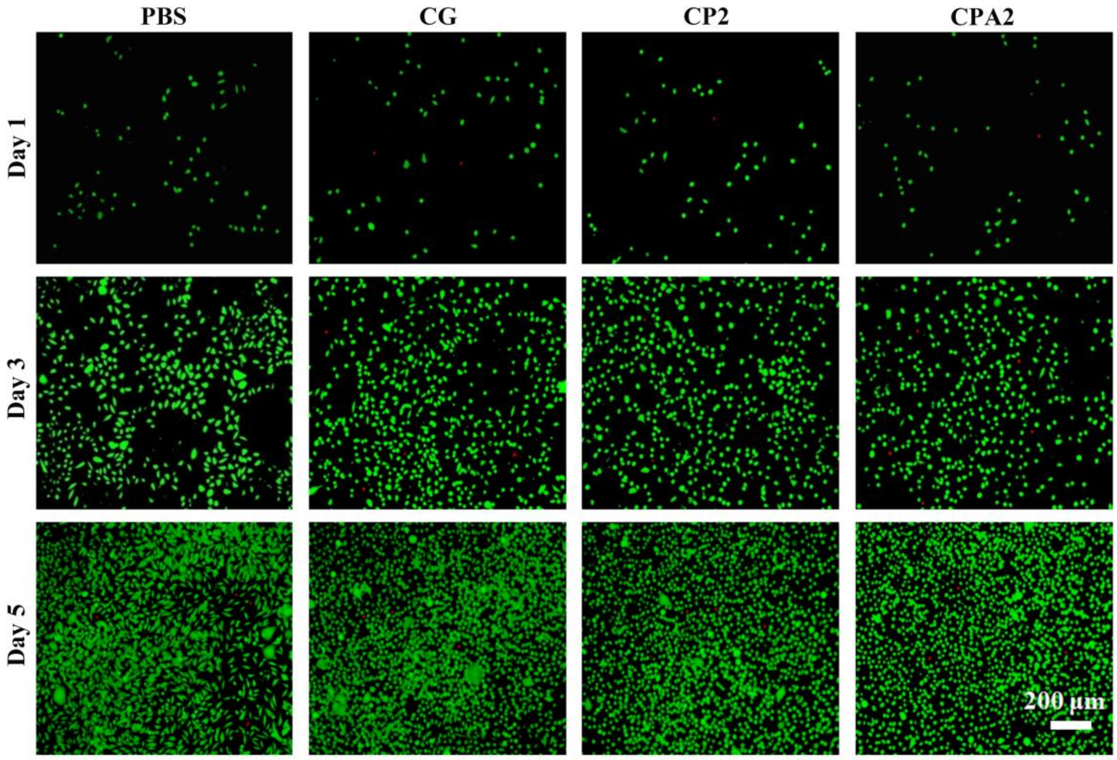

Figure S19. Fluorescent images of calcein-AM and PI double-stained L929 cells after treating with different samples for 1 day, 3 days, and 5 days.

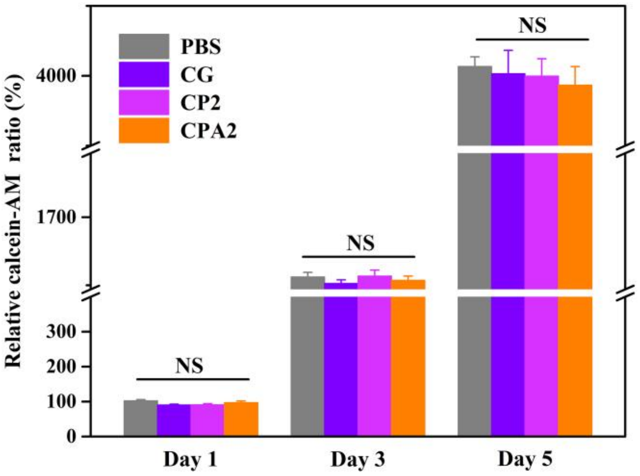

Figure S20. Relative calcein-AM fluorescence ratio of L929 cells treated with different samples for 1 day, 3 days, and 5 days (n = 3). NS means not significant.

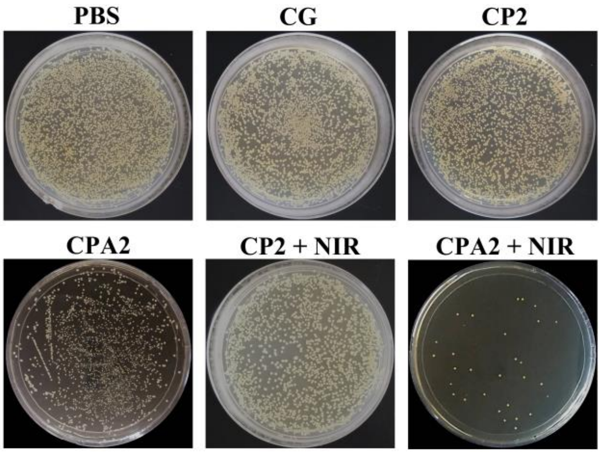

Figure S21. Photographs of bacterial colonies on agar plates from wound sites on the first day after different treatments (n = 3).

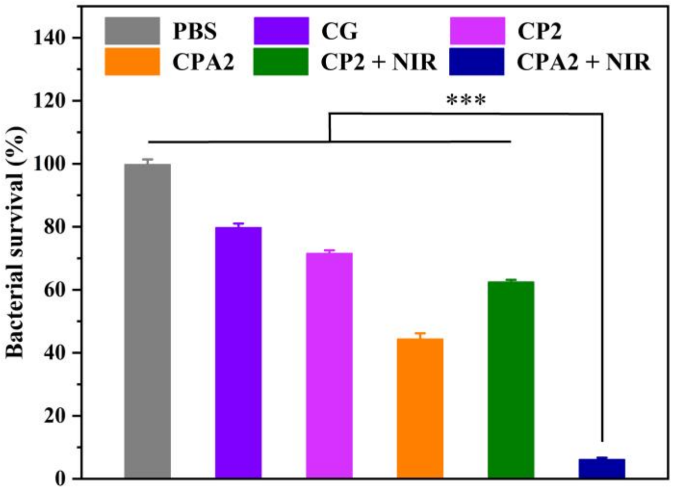

Figure S22. Quantitative analysis of bacterial colonies on agar plates from wound sites on the first day after different treatments (n = 3). *** means P < 0.001.

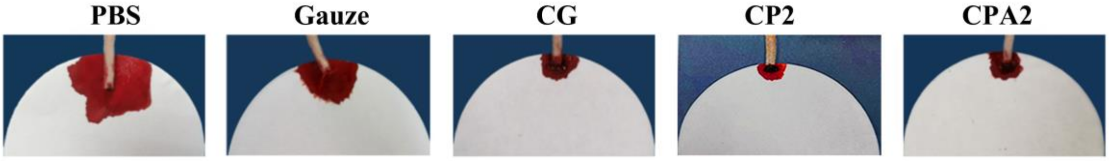

Figure S23. Representative images demonstrating hemostasis after creating an incision in the tail of a rat (n = 3).

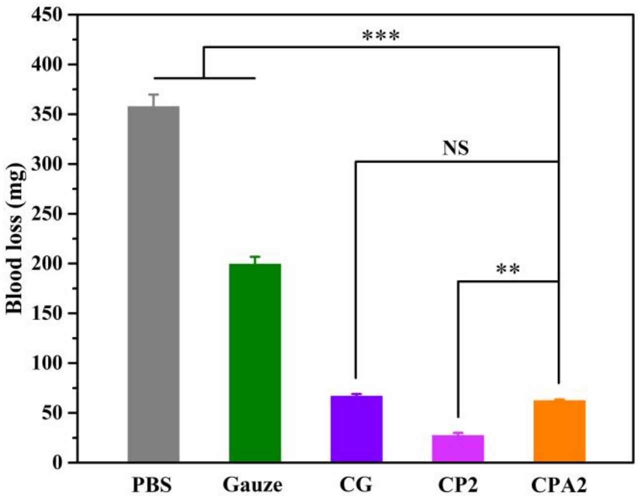

Figure S24. Blood loss of PBS, gauze, CG, CP2 and CPA2 in the rat-tail amputation model (n = 3). ** means P < 0.01, *** means P < 0.001 and NS means not significant.

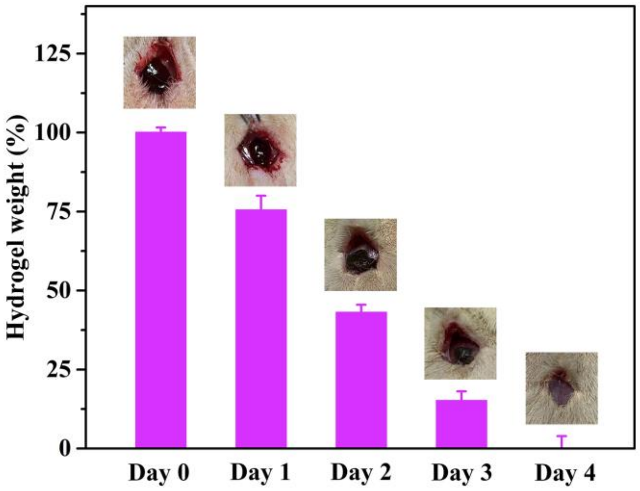

Figure S25. *In vivo* degradation process of CPA2 hydrogel (n = 3).

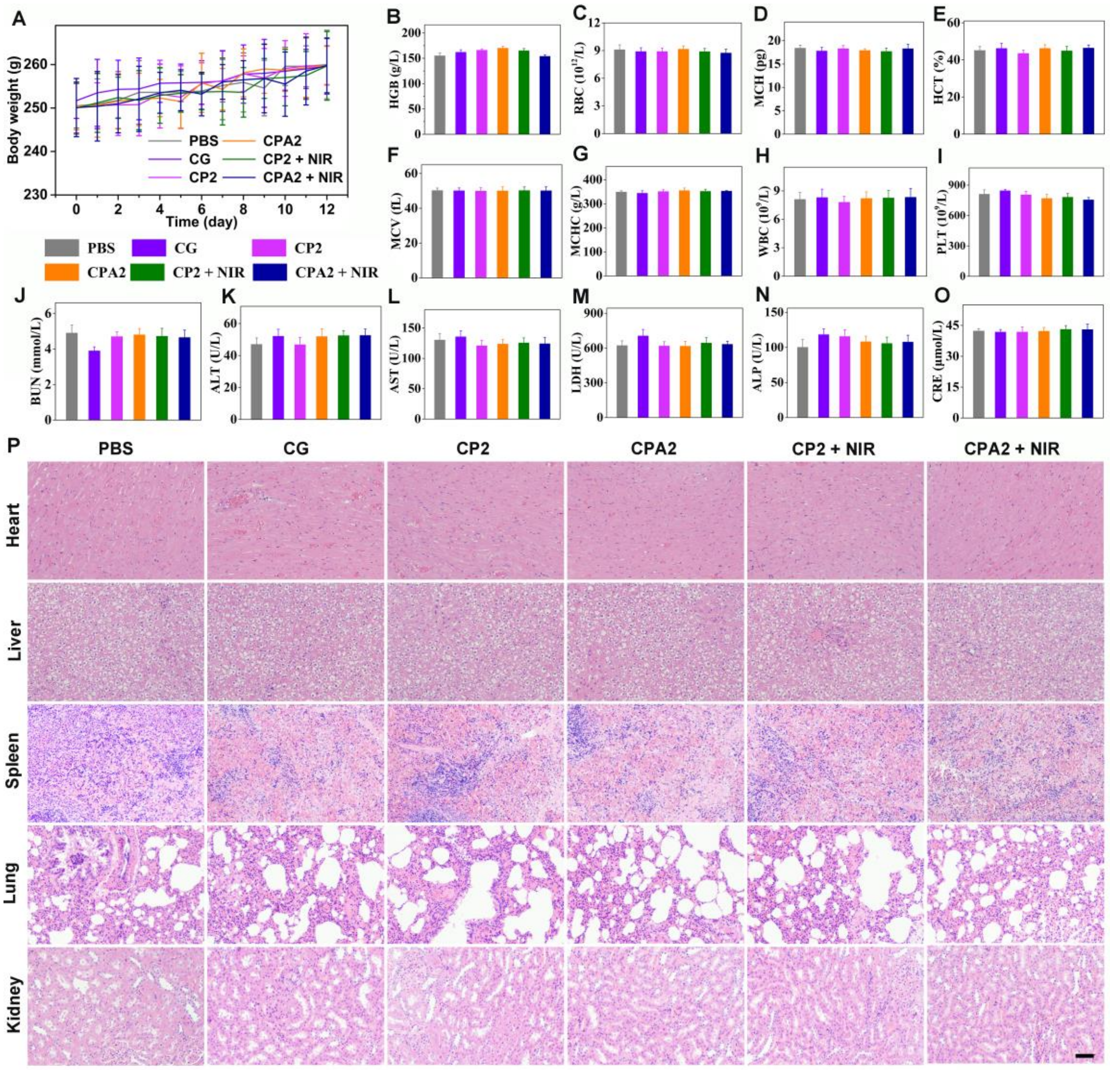

Figure S26. Preliminary toxicity assay. (A) Body weight changes of rats during different treatments. (B–O) Blood analysis of the rat at the end of treatment: (B) hemoglobin (HGB), (C) red blood cells (RBC), (D) mean corpuscular hemoglobin (MCH), (E) hematocrit (HCT), (F) mean cell volume (MCV), (G) mean corpuscular hemoglobin concentration (MCHC), (H) white blood cells (WBC), (I) blood platelet (PLT), (J) blood urea nitrogen (BUN), (K) alanine transferase (ALT), (L) aspartate transferase (AST), (M) lactate dehydrogenase (LDH), (N) alkaline phosphatase (ALP) and (O) creatinine (CRE). (P) H&E staining of main organs (heart, liver, spleen, lung, and kidney) collected from rats in the different groups. Scale bar = 50 μm.

References

[1] Q. Zeng, Y. Qian, Y. Huang, F. Ding, X. Qi, J. Shen, *Bioact. Mater.* **2021**, 6, 2647.

[2] N. Zandi, E. S. Sani, E. Mostafavi, D. M. Ibrahim, B. Saleh, M. A. Shokrgozar, E. Tamjid, P. S. Weiss, A. Simchi, N. Annabi, *Biomaterials* **2021**, 267, 120476.

[3] Y. Hu, L. Barbier, Z. Li, X. Ji, H. L. Blay, D. Hourdet, N. Sanson, J. W. Y. Lam, A. Marcellan, B. Z. Tang, *Adv. Mater.* **2021**, 2101500.

[4] D. Humelnicu, M. M. Lazar, M. Ignat, I. A. Dinu, E. S. Dragan, M. V. Dinu, *J. Hazard. Mater.* **2020**, 381, 120980.

[5] X. Qi, T. Su, M. Zhang, X. Tong, W. Pan, Q. Zeng, Z. Zhou, L. Shen, X. He, J. Shen, *ACS Appl. Mater. Interfaces* **2020**, 12, 13256.

[6] G. Liu, Z. Bao, J. Wu, *Chinese Chem. Lett.* **2020**, 31, 1817.

[7] C. Chen, Y. Wang, D. Zhang, X. Wu, Y. Zhao, L. Shang, J. Ren, Y. Zhao, *Appl. Mater. Today* **2021**, 23, 101000.
